# Recent advances in understanding the role of leptin in energy homeostasis

**DOI:** 10.12688/f1000research.24260.1

**Published:** 2020-05-27

**Authors:** Heike Münzberg, Prachi Singh, Steven B. Heymsfield, Sangho Yu, Christopher D. Morrison

**Affiliations:** 1 Pennington Biomedical Research Center, Louisiana State University System, Louisiana, USA

**Keywords:** Food intake, energy expenditure, Arcuate nucleus, dorsomedial hypothalamus, MC4R, POMC, AgRP, neuronal activity, fiber photometry

## Abstract

The hormone leptin plays a critical role in energy homeostasis, although our overall understanding of acutely changing leptin levels still needs improvement. Several developments allow a fresh look at recent and early data on leptin action. This review highlights select recent publications that are relevant for understanding the role played by dynamic changes in circulating leptin levels. We further discuss the relevance for our current understanding of leptin signaling in central neuronal feeding and energy expenditure circuits and highlight cohesive and discrepant findings that need to be addressed in future studies to understand how leptin couples with physiological adaptations of food intake and energy expenditure.

## Introduction

The adipose tissue-derived hormone leptin plays a critical role in whole body energy homeostasis: lack of functional leptin or leptin receptors (Lepr) results in severe and early onset obesity in rodents and humans, while leptin replacement in leptin-deficient rodents and humans fully recovers energy homeostasis ^1, 2^. Leptin mediates its effect via the central nervous system, and neuron-specific re-expression of Lepr in whole body Lepr-deficient mice is sufficient for full recovery of all known physiological effects of leptin and Lepr deficiency ^3^. Nevertheless, documented peripheral leptin action might support several aspects of leptin functions ^4– 6^.

A more confusing literature addresses leptin’s role in normal physiology and the relevance of the observed dynamic changes in circulating leptin levels. Long-term circulating leptin levels are positively correlated with adiposity, but short-term circadian changes ^7, 8^ or acute changes (hours) in response to physiological challenges such as fasting/refeeding ^9, 10^, cold exposure ^11^, sleep restriction ^12– 14^, hypoxia ^15, 16^, methionine restriction ^17, 18^, or type 1 diabetes ^9^ are known.

Circulating leptin levels reflect leptin sensitivity with regard to the ability of exogenous applied leptin to increase signaling pathways and physiological function. A drop in leptin levels sensitizes while high leptin levels blunt leptin signaling and function, also known as leptin resistance. This resistance is mediated via negative feedback signals in the Lepr signaling cascade that build up with elevated leptin levels (or diminish with low leptin levels) and suppress leptin signaling efficiency (for detailed reviews, see 19– 22).

Yet there is no consensus on how changes in endogenous leptin levels are relevant for body weight homeostasis. High leptin levels in obesity are particularly puzzling because leptin seems to be unable to reduce food intake and prevent obesity, despite appropriate induction of many early leptin signaling events ^23^. Some data even suggest that hyperleptinemia may prevent further detrimental effects on food intake, body weight, and glucose homeostasis ^24– 26^. Weight loss that is achieved with dieting and exercise is “sensed” as negative energy balance and the associated drop in leptin levels enables physiological adaptations (e.g. decreased energy expenditure and increased hunger) geared to favor weight regain. These adaptations are reversed by leptin treatment, which improves weight loss maintenance in mice and humans ^27– 29^. Conversely, the dramatic weight loss achieved with bariatric surgery is surprisingly not “sensed” as an energy need state, compared to weight-matched control animals, despite similarly decreased leptin levels ^30^. The lack of adaptive responses enables the stunning long-term weight loss and improved glucose homeostasis following bariatric surgery. A critical future task is to understand how dynamic changes in leptin levels couple with (or uncouple from) physiological adaptations of food intake and energy expenditure.

This review will highlight some recent publications that are relevant for the role of dynamic changes in circulating leptin levels in health and disease. We further discuss their relevance for our current understanding of leptin signaling in central feeding and energy expenditure circuits and highlight cohesive and discrepant findings that need to be addressed in future studies to understand how leptin couples with physiological adaptations.

## Regulation of circulating leptin levels and functional importance

Leptin levels are regulated by a feedback signal according to the body’s energy availability ^31^. Inhibition of leptin gene expression is generally associated with a negative energy state or increased energy need states, such as fasting ^11^, cold exposure ^10^, methionine restriction ^17, 18^, type 1 diabetes ^9, 32^, or exercise ^33^. Neuroendocrine adaptations to these physiological conditions, such as fasting (e.g. increased corticosterone and ACTH, low thyroid hormone), can be reversed or significantly blunted when leptin levels are restored ^27, 29^. Conversely, increased leptin gene expression is associated with a positive energy state, and circulating leptin correlates positively with adipose tissue mass ^34^.

However, the key regulatory processes that control leptin gene expression remain unclear. Only recently, a novel PPARγ/RXR binding site was identified within one of the leptin promoter elements that also restricts leptin gene expression to adipose tissue. These data suggested the binding of PPARγ with one or more unidentified factor(s) that together enable the suppression of leptin gene expression and adipose tissue lipolysis may likely induce this factor ^35^. Adipose tissue lipolysis is a hallmark of energy need states and depends on β3-adrenergic receptor signaling. Similarly, leptin gene expression is suppressed by increased sympathetic tone via β3-adrenergic receptors ^10, 11, 36– 38^.

Recent work also found that low leptin levels are required to increase the HPA axis ^9, 32^, even though this view is debated ^39^. This mechanism is intriguing, as increased glucocorticoids are associated with many hypoleptinemic states like fasting, sleep deprivation, and cold exposure ^9, 12, 40^. This emerging role of glucocorticoids to drive hunger and food intake in response to decreased leptin levels ^9, 41, 42^ might be critical to understand the coupling of leptin levels with physiological adaptations of feeding and energy expenditure. However, the mechanisms through which central circuits regulate leptin gene expression are unclear. Such circuits will likely control sympathetic tone in adipose tissue and have been described for brown adipose tissue (BAT) ^43, 44^ and are likely distinct from white adipose tissue (WAT) based on the anatomical dissociation of pre- and post-ganglionic inputs to BAT and WAT 44 and Huesing *et al.* unpublished data). Also, some studies suggested distinct contribution of subsets of arcuate nucleus (ARC) neurons for differential sympathetic tone to adipose tissues, substrate fluxes, and suppression of leptin gene expression ^45– 47^.

## Central leptin action and energy homeostasis

Central hypothalamic circuits are critical to mediate leptin signaling and promote energy homeostasis via modulation of food intake and energy expenditure. Within the ARC, two conversely acting neuronal populations are well characterized for their interaction with leptin: anorexigenic and energy expenditure-inducing pro-opiomelanocortin (POMC) neurons and orexigenic and energy expenditure-suppressing agouti-related peptide (AgRP) neurons. POMC-derived peptides have stimulatory and AgRP inhibitory effects on melanocortin-4 receptors (MC4R) ^48, 49^, and their converse actions may involve several levels (e.g. antagonism, Gi/Gs signaling) ^50^. Changes in neuronal activation of POMC and AgRP neurons are critical for sensing energy availability states, and both populations are responsive to a range of orexigenic and anorexigenic signals ^51^. AgRP neurons are activated during energy need states (low leptin levels), while POMC neurons are activated in energy replete or overfeeding states (normal/high leptin levels). Initially, this correlation was histologically shown by expression of the early response gene *cFos*, as well as POMC and AgRP, which correlated well with neuronal activation ^48, 52, 53^; recent studies confirmed this with fiber photometry, which allowed real-time observation of neuronal activity changes ^54, 55^. AgRP neuronal activation studies further indicated that feeding behavior dissociates into rapid (within minutes) and slow (within days) feeding events ^56– 58^. Generally, AgRP neuron-induced rapid feeding events require the co-expressed neuropeptide NPY ^56, 59, 60^, while slow feeding events require AgRP or POMC signaling via MC4R ^52, 60, 61^.

The temporal responses of AgRP and POMC neurons are particularly relevant for leptin signaling. Recent data demonstrated a gradual and slow leptin action in POMC and AgRP neurons, while rapid activity changes are mediated by many gut peptides, but not leptin ^51^. The slow kinetics of leptin action are consistent with transcriptional events and in line with many early studies demonstrating that leptin induces POMC mRNA and suppresses AgRP mRNA (reviewed in 48). Importantly, the weight loss achieved with bariatric surgery suppresses leptin levels similar to what is seen in weight-matched animals, but the increased AgRP mRNA was absent with bariatric surgery, unlike in weight-matched animals ^62^. Similarly, MC4R-deficient and leptin-deficient mice are resistant or show blunted weight loss to bariatric surgery ^63, 64^, further pointing to an important role for leptin>MC4R signaling to mediate this effect, even though sleeve gastrectomy-mediated weight loss did not depend on MC4R signaling ^65^.

Thus, a better understanding of how leptin modulation of MC4R signaling integrates into neuronal circuits that regulate feeding and energy expenditure would be important to target unwanted coupling of low leptin levels with physiological adaptations in obese individuals as well as beneficial uncoupling in bariatric surgery patients.

### Energy homeostasis and food intake

**Figure 1.**
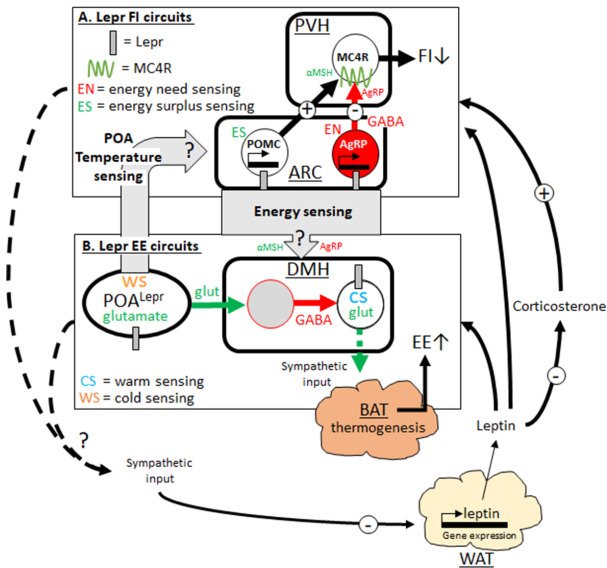
Lepr-centric scheme of feeding and energy expenditure circuits. Changing leptin levels act on food intake (FI, 1A) and energy expenditure (EE, 1B) via divergent MC4R neurons in the paraventricular hypothalamus (PVH) ^68^ and dorsomedial hypothalamus (DMH) ^72^, respectively. Temperature sensing via the preoptic area (POA) and energy sensing via the arcuate nucleus (ARC) are both associated with changing leptin levels and require physiological adaptations of FI and EE. Both sensory inputs are likely to integrate via the same FI and EE circuits. Recent data further suggest that low leptin levels enable agouti-related peptide (AgRP)-induced feeding via an increased HPA axis ^9^, raising awareness for a tight interaction of peripheral and central signaling systems. Dynamic changes in leptin levels are a critical part in these FI and EE circuits, and the central feedback mechanisms for this important link are unclear. α-MSH, α-melanocyte-stimulating hormone; BAT, brown adipose tissue; GABA, gamma aminobutyric acid; Lepr, leptin receptor; MC4R, melanocortin-4 receptor; POMC, pro-opiomelanocortin; WAT, white adipose tissue.

Leptin levels are critical to regain energy homeostasis following fasting or overfeeding. Leptin acts via POMC and AgRP neurons to suppress MC4R neurons in the paraventricular hypothalamus (PVH) ^29, 66– 68^, which can be considered the critical circuit to couple changes in leptin levels with food intake adaptations ( Figure 1A). However, fasting-induced hyperphagia or overfeeding-induced hypophagia cannot be explained by acute neuronal activation states of POMC and AgRP neurons. The fasting-induced activity patterns in POMC and AgRP neurons (POMC activity ↓, AgRP activity ↑) normalize within seconds of food availability ^55^, so that the observed long-lasting hyperphagia must be mediated by additional mechanisms.

Low leptin causes chronic cFos expression in AgRP neurons and is thought to reflect chronic activation of AgRP neurons. Thus, leptin-deficient *ob/ob* mice might not respond properly to refeeding and fail to normalize AgRP neuronal activity. Surprisingly, POMC and AgRP neurons in *ob/ob* mice remain fully responsive ^51^, concluding that acute activity changes are not the main cause for obesity in *ob/ob* mice. Instead, these data refocus our attention to earlier studies, demonstrating the importance of transcriptional changes in AgRP and POMC mRNA for weight gain and obesity ^69^. Similarly, long-term feeding and AgRP expression can be mediated independent of neuronal activity and require at least one additional transcription factor ^52^, even though it is currently unclear if slow-acting leptin, rapid-acting gut peptides, or associated factors like corticosterone may have the same or distinct impact on melanocortin gene expression.

**Figure 2.**
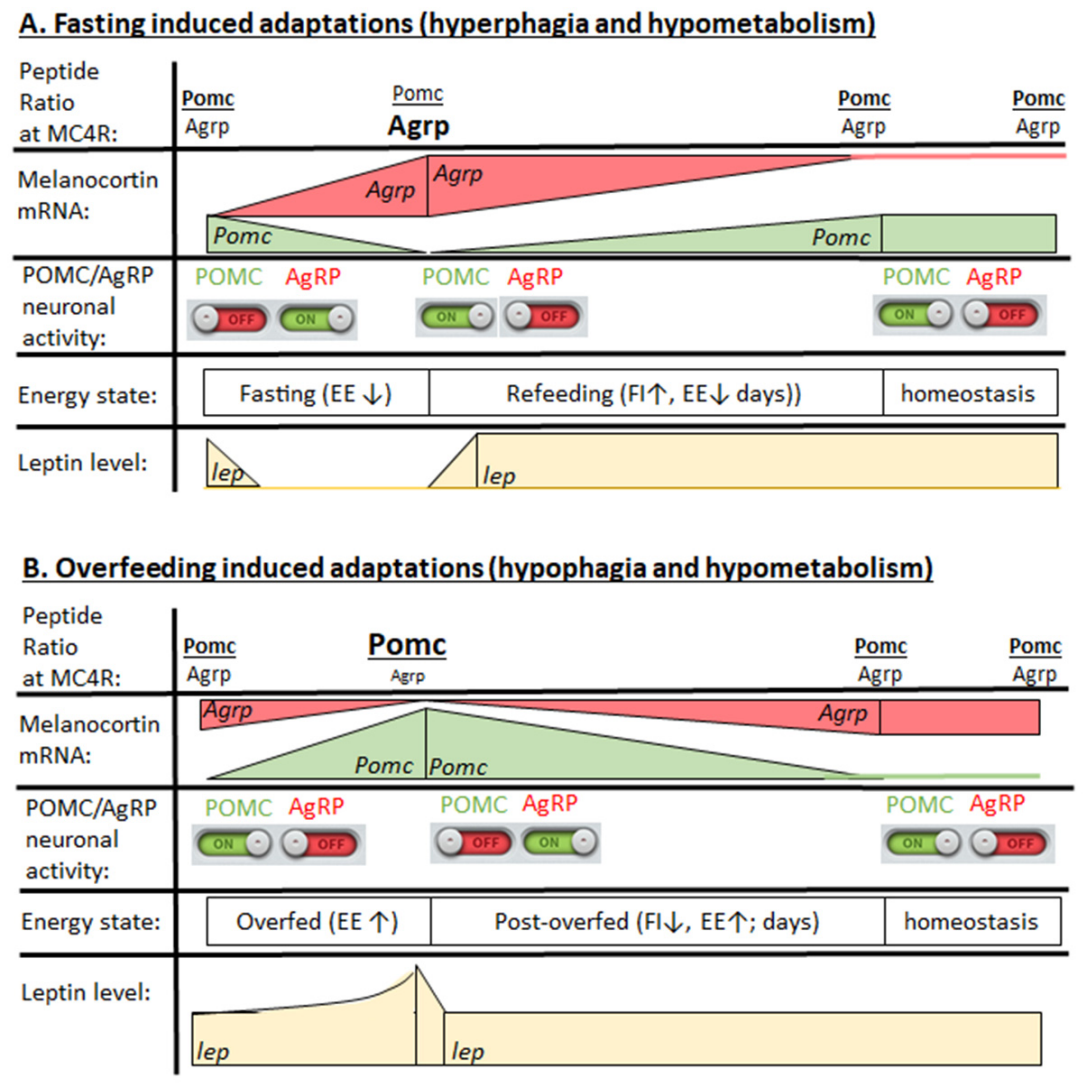
Schematic diagram to explain how acute changes in POMC and AgRP neuronal activity can be dissociated from the dynamic changes in leptin levels and their interaction with POMC and AgRP gene expression and melanocortin peptide ratios to stimulate or inhibit MC4Rs in food intake and energy expenditure circuits. Note, the duration and fold change of leptin mRNA expression as well as release into the serum have not been fully explored, but a full suppression of leptin mRNA within 2 hours has been reported ^10^. AgRP, agouti-related peptide; EE, energy expenditure; FI, food intake; POMC, pro-opiomelanocortin; MC4R, melanocortin-4 receptor.

We speculate that the duration of fasting or overfeeding shifts the ratio of POMC-derived peptides (energy surplus) versus AgRP peptide (energy need) and has prolonged effects on MC4R signaling. Dependent on the stability of mRNA and peptides, this system could be active despite normalized neuronal activity and explains why physiological adaptations to favor weight gain are long-lasting in contrast to the rapid neuronal activation changes. Indeed, the duration of AgRP neuronal activity correlates with the amount of food ingested ^55^, but this study did not specifically study long-term feeding. AgRP is sufficient to mediate long-term feeding responses ^58, 60^, even though rapid changes in feeding via NPY may contribute to overall food intake. Conversely, rapid normalization of AgRP and POMC neuronal activity and leptin levels would promote normalization of POMC/AgRP ratios over time to reinstate homeostasis ( Figure 2).

In line with this concept, lack of functional POMC expression (causing unopposed AgRP action) causes severe obesity with increased food intake and suppressed energy expenditure ^70^. Conversely, prolonged treatment with an MC4R agonist was able to promote lasting weight loss in Lepr-deficient patients ^71^. Thus, re-establishing the energy surplus signal from POMC neurons seems to be a key component for long-lasting weight loss. Similarly, POMC neurons require chronic (24 hours) optogenetic activation to suppress feeding, which was blocked in *Agouti* mice with chronic MC4R inactivation ^61^ and is consistent with a slow buildup of POMC-derived peptides at MC4R neurons. Earlier studies also showed that elevated POMC expression and MC4R signaling are important mediators of the homeostatic adaptations to overfeeding and these adaptations were prevented by blocking MC4R with an antagonist ^66^ or in leptin signaling-deficient animals ^67^. These observations would explain why bariatric surgery is ineffective in MC4R knockout or *ob/ob* mice and suggests a prominent role for MC4R signaling in the full beneficial effects of bariatric surgery.

Furthermore, the role of leptin signaling in ARC neurons has been confusing following initial work which removed Lepr from POMC, AgRP, or both neurons ^73, 74^. The observed mild effects on body weight compared to whole body Lepr-deficient *db/db* mice led to a shift away from an ARC-centric view of leptin action, and several non-ARC Lepr neurons were discovered with similarly mild contributions to whole body leptin function ^75^. However, a recent study used CRISPR technology for an acute and AgRP-specific Lepr deletion ^42^ and clarified that AgRP-specific Lepr deletion explained ~80% of the obese phenotype in *db/db* mice. This effect was largely independent of direct changes in neuronal activity, again suggesting that transcriptional events (increased AgRP mRNA) may most prominently drive this effect. This study also deleted Lepr from POMC neurons with only mild effects on body weight and food intake, even though a thorough investigation of homeostatic adaptations in response to overfeeding was not performed ^42^.

Together, these studies again reinforce the importance of ARC Lepr neurons in maintaining body weight homeostasis, yet the relative contribution of POMC versus AgRP neurons needs re-clarification, specifically since recent studies show a strong bias towards the importance of AgRP versus POMC neurons. New state-of-the-art methods such as fiber photometry to study the long-term effects of leptin and melanocortin signaling on neural activity are problematic, since this method is mainly used to observe acute activity changes. However, long-term activity changes were successfully observed in leptin-treated *ob/ob* mice, even though these responses could not be observed in wild-type mice ^51^. A recent fiber photometry study in PVN MC4R feeding neurons surprisingly failed to show feeding-induced activity changes ^76^, possibly because only acute responses were evaluated. Thus, future development of experimental paradigms that allow reliable interpretation of long-term effects of leptin and melanocortin signaling on neuronal activity and feeding responses is critical to connect new technologies with this important signaling pathway in energy homeostasis.

### Energy homeostasis and energy expenditure

The role of leptin in increasing energy expenditure has been debated ^77^ in contrast to the well-accepted anorexigenic leptin actions. Leptin increases the sympathetic tone to BAT, a site of increased energy usage and thus increased energy expenditure ^78, 79^. However, leptin injections are unable to influence metabolic rate *per se* ^80, 81^. Instead, leptin counteracts the hypometabolism induced by energy need states in both rodents and humans ^27, 28, 80, 82^. Furthermore, leptin affects thermoregulation, body temperature, and cold sensitivity in mice ^83– 86^, even though this has not been observed in humans ^1^.

Lepr-expressing neurons are part of a thermoregulatory circuit that connects the preoptic area (POA) and dorsomedial hypothalamus/dorsal hypothalamic area (DMH/DHA) to the sympathetic control of BAT ^87^. Lepr neurons in the DMH/DHA explain many leptin functions on energy expenditure and body temperature. DMH/DHA Lepr neurons are activated by cold exposure (cold-sensing neurons) and leptin ^43, 85^, and activation of DMH/DHA Lepr neurons is sufficient to increase energy expenditure via sympathetic activation of BAT that depends on β3-adrenergic signaling. Lepr expression in these neurons is required to prevent hypometabolism and weight gain, and DMH/DHA leptin infusion is sufficient to normalize body temperature and improve body weight in *ob/ob* mice and obese rats without affecting food intake ^43, 88^. Together, these observations suggest that DMH/DHA Lepr neurons are important for coupling changes in leptin levels with energy expenditure adaptations ( Figure 1B).

Lepr neurons in the POA are a homogenous population of glutamatergic neurons that are activated by warm temperature (warm-sensing neurons), and chemogenetic activation causes a robust suppression of energy expenditure ^89^. Originally, warm-sensing POA neurons were identified as GABAergic neurons, based on the marker glutamate decarboxylase (Gad1/2 aka Gad65/67) ^90, 91^. However, detailed single cell profiling of POA neurons clarified its expression in both GABA and glutamatergic neurons and ultimately verified warm-sensing POA neurons as a homogenous glutamatergic cluster with Lepr expression ^92^. Warm-sensing POA neurons innervate the DMH and inhibit cold-sensing DMH/DHA neurons ^43, 89, 91, 93– 95^, likely indirectly via GABAergic interneurons ( Figure 1B).

Leptin signaling has no effect on temperature-dependent energy expenditure changes ^81, 96^, but POA leptin action surprisingly contributed to counteracting weight gain in a state of energy surplus (high-fat diet feeding) and energy expenditure adaptations in an energy need state (fasting-induced hypometabolism) ^81^. This suggested that POA Lepr neurons play a role in coupling leptin signaling with energy expenditure adaptations. As noted above, negative and positive energy states are classically controlled by ARC POMC (energy surplus sensing) and AgRP neurons (energy need sensing), including acute changes in neuronal activity as well as transcriptional changes of POMC and AgRP expression. Thus, an interaction of temperature-sensing POA Lepr neurons with energy-sensing mechanisms suggests that both sensory inputs merge into the same circuits. Interestingly, a recent study demonstrated that the MC4R agonist MTII induces energy expenditure exclusively via DMH MC4R ^72^. Importantly, DMH MC4R had no effect on the food intake-suppressing effects of MTII ^72^, which is mediated exclusively by PVN MC4R neurons ^68^. Thus, these data further indicate the DMH as the likely integration site for ARC and POA neurons on energy expenditure adaptations ( Figure 1B).

Warm-sensing POA neurons also suppress food intake in response to ambient temperature, and activation of warm-sensing POA Lepr neurons robustly reduces body weight ^81, 97^. Interestingly, leptin-deficient mice are unable to suppress their food intake in response to warm ambient temperature, and their food intake is maintained at levels comparable to cold-exposed animals ^96^. The hyperphagia in *ob/ob* mice is caused by the unopposed AgRP action at PVN MC4R neurons and is resistant to acute POMC activation ^61^. Thus, we speculated that temperature-induced food intake adaptations also integrate into the same feeding circuit as energy-sensing inputs (PVN MC4R neurons) ( Figure 1A).

An integration of temperature and energy sensing via distinct feeding and energy expenditure circuits may further integrate other sensory inputs associated with changes in leptin levels (sleep restriction, hypoxia, protein restriction, etc.), even though integration could also occur downstream to the PVN and DMH within these circuits.

## Summary and outlook

Recent work has identified leptin promoter regions that mediate dynamic changes in leptin levels and predicts binding factors that are activated by lipolysis to suppress leptin levels. We further emphasized the importance of understanding the physiological cause and function of leptin levels in healthy individuals: to understand how we can leverage this system to treat metabolic dysfunctions. We consider the coupling of leptin levels with physiological adaptations of food intake and energy expenditure as critical components to treat metabolic dysfunction because they prevented long-term weight maintenance in obese patients and their uncoupling enables long-term weight loss in bariatric surgery patients. We make the case for an emerging concept that couples leptin signaling with food intake adaptations via PVN MC4R neurons and with energy expenditure adaptations via DMH MC4R neurons. We outline evidence indicating substantial integration of temperature- and energy-sensing adaptations of food intake and energy expenditure across these circuits. These concepts are likely relevant for other non-metabolic leptin functions, such as reproduction, that we did not specifically discuss in this review. Finally, we highlight the need to develop new protocols for current state-of-the-art technologies to accommodate the study of slower kinetics for leptin and melanocortin signaling to ensure future progress in this field.
